# Mr. Harrup's Observations on Acute Rheumatism

**Published:** 1811-12

**Authors:** Robert Harrup

**Affiliations:** Chobham


*To the Editors of the Medical and Physical Journal.*

GENTLEMEN,

I TAKE the liberty of sending you a few observations on a remedy for Acute Rheumatism, which is recommended by Mr. Davies in your last number.

Practitioners have long been in the habit of administering opium with calomel in various diseases, merely with the view of either relieving pain or of preventing the latter from passing off too quickly by the bowels. Dr. Hamilton, of Lynn-Regis, was, however, the first who pointed out the superior efficacy of these medicines, when conjoined in inflammatory affections,† after a successful employment of them for sixteen years. The general mode in which that gentleman administered the remedy was as follows: In the beginning of the disease he directed blood to be taken away, proportioned to the violence of the inflammatory symptoms and the constitution of the patient. The bowels were then emptied by a clyster, or more frequently by a purgative. Afterwards one

† Vide Edin. Medical Commentaries, Vol. 9.

 grain grain of calomel, and from a fourth to a grain of opium in the form of a bolus, were administered every six, eight, or twelve hours, as the degree of inflammation or the threatening appearance of the disorder seemed to require.

Dilution with barley-water, &c. was at the same time strictly enjoined. After taking three or four doses of the medicine in the course of 24 hours, the patient was generally relieved; and in 24 more the distemper commonly gave way and soon terminated. When not relieved in the first 24 hours, and the high inflammatory symptoms continued with little or no abatement (which was rarely the case), more blood was taken away, and the mercurial composition directed to be more frequently taken, and continued until the disease resolved either by sweating, purging, or more commonly both, or by a ptyalism being raised.

He further observes, that if this method of cure was employed early in the disease, the patient was soon relieved, whatever was the operation of the mercury; but if employed late it was attended with more uncertainty, the case was rendered more doubtful, and the recovery more slow, but most commonly *the soonest when the salivary glands were affected.* When the skin was dry and contracted he added tartarized antimony, and sometimes camphor, to the composition; and observes, that he never found any medicine, either in a simple or aggregate state, produce so certainly, speedily, and effectually, a relaxation of the skin and a plentiful discharge from its pores, as a composition of calomel, tartarized antimony, opium, and camphor, which has also the advantage of increasing the evacuations by stool and urine.

Although, since Dr. Hamilton's recommendation, several practitioners have employed a composition of calomel and opium in such cases with great benefit, it is somewhat surprizing that the remedy has never come into general practice. From many years experience of its efficacy in the generality of acute inflammatory diseases, I consider it as superior to any other remedy with which we are acquainted in all such cases. If taken at the commencement of acute rheumatism, it checks the inflammatory symptoms in the course of twenty-four hours, and relieves the excruciating pains. At the same time it must be acknowledged, that in many cases it will not perform a cure unless the *mouth be affected by the mercury.*

In pneumonia it is a most valuable remedy; I have found it in a number of cases supersede the necessity of blood-letting, and consequently avoid the debility which taking away a quantity of blood never fails to produce, and which superadded to that debility, which is the natural consequence of the the disease, too frequently lays the foundation of fatal chronic complaints.

In enteritis it will be found equally powerful; and in cases of gout, when attacking young plethoric habits, it is of very essential service in abating the inflammation and pain, particularly if the calomel is so proportioned as to freely affect the bowels. In short, if this remedy is properly and early administered in any of those diseases where acute inflammation is present, it will seldom, if ever, disappoint the expectations of the practitioner. I must, therefore, join with Mr. Davies in calling the attention of the medical world to a remedy so important in its effects. Some years ago an epidemic of a very singular nature and fatal tendency prevailed in this neighbourhood, (an account of which I may at some future period send you); the symptoms at the commencement were violently inflammatory, which in every case were completely removed by the exhibition of calomel and opium.

From long observation I am inclined to think with Mr. Davies, that the two medicines given separately will not produce the same effect as when combined. Opium is generally admitted to be injurious in acute inflammation, and calomel is never given with the view of mitigating the disease. What I conceive to be the most convincing proof of the composition being specifically efficacious, is, that I have very often observed the inflammatory symptoms greatly abated long before any evacuation whatever took place; Dr. Hamilton also makes the same remark.

In the case recorded by Mr. Davies the mouth was slightly affected with the calomel, which, I consider, renders the *composition* very doubtful. In that case I am convinced, from long experience, that if calomel or any other preparation of mercury had been administered alone till the mouth had been affected, the disease would either have been suspended or eradicated. To enlarge on this subject is unnecessary, having already delivered my sentiments in a former volume of the Medical Journal.

I am, Gentlemen,
Your obedient humble Servant,
ROBERT HARRUP.

*Chobham, Sept.* 25, 1811.

P. S. Your correspondent, Medicus, who desires to be informed of the process for dissolving elastic gum in æther, will find a minute account in the second vol. of your Journal, page 83; and some further particulars respecting the different solvents of that substance, in a letter inserted in the 17th volume, page 454.

